# Metabolic phenotyping for monitoring ovarian cancer patients

**DOI:** 10.1038/srep23334

**Published:** 2016-03-21

**Authors:** Chaofu Ke, Ang Li, Yan Hou, Meng Sun, Kai Yang, Jinlong Cheng, Jingtao Wang, Tingting Ge, Fan Zhang, Qiang Li, Junnan Li, Ying Wu, Ge Lou, Kang Li

**Affiliations:** 1 Department of Epidemiology and Biostatistics, School of Public Health, Harbin Medical University, Harbin 150086, P.R. China; 2 Department of Cardiology, The Second Affiliated Hospital of Harbin Medical University, and The Key Laboratory of Myocardial Ischemia, Harbin Medical University, Harbin 150086, P.R. China; 3 Department of Gynecology Oncology, The Tumor Hospital, Harbin Medical University, Harbin 150086, P.R. China

## Abstract

Epithelial ovarian cancer (EOC) is the most deadly of the gynecological cancers. New approaches and better tools for monitoring treatment efficacy and disease progression of EOC are required. In this study, metabolomics using rapid resolution liquid chromatography mass spectrometry was applied to a systematic investigation of metabolic changes in response to advanced EOC, surgery and recurrence. The results revealed considerable metabolic differences between groups. Moreover, 37, 30, and 26 metabolites were identified as potential biomarkers for primary, surgical and recurrent EOC, respectively. Primary EOC was characterized by abnormal lipid metabolism and energy disorders. Oxidative stress and surgical efficacy were clear in the post-operative EOC patients. Recurrent EOC patients showed increased amino acid and lipid metabolism compared with primary EOC patients. After cytoreductive surgery, eight metabolites (e.g. l-kynurenine, retinol, hydroxyphenyllactic acid, 2-octenoic acid) corrected towards levels of the control group, and four (e.g. hydroxyphenyllactic acid, 2-octenoic acid) went back again to primary EOC levels after disease relapse. In conclusion, this study delineated metabolic changes in response to advanced EOC, surgery and recurrence, and identified biomarkers that could facilitate both understanding and monitoring of EOC development and progression.

Epithelial ovarian cancer (EOC) continues to be one of the most frequently diagnosed malignancies worldwide, and is the most deadly of the gynecological cancers^1^. The majority of patients tend to present in advanced stages, and 5-year survival rates are below 30%^2^. Standard treatment for advanced EOC involves cytoreductive surgery followed by platinum-based chemotherapy^3^. Despite the favorable response to initial therapy, most patients will relapse within 18 months^3^. Recurrent EOC usually develops chemotherapy resistance and invariably is fatal^4^. To improve survival rates, new efforts should be devoted to the discovery of novel biomarkers to monitor disease progression and personalize therapy for advanced EOC patients.

Cancer cells possess a fundamentally altered metabolism that enables tumorigenicity and malignancy^5^. Our understanding of the biochemical underpinnings of cancer development and progression could lead to the discovery of potential therapeutic targets and the achievement of individualized disease management. Metabolomics, which is defined as the global measurement of endogenous metabolites in a given biological sample, provides comprehensive insights into biological processes^6^. Over the past decade, metabolomics has been successfully applied to studies of cancer metabolism in various cancers, including ovarian cancer^7^,^8^,^9^,^10^,^11^,^12^,^13^,^14^. Our previous plasma and urinary metabolomic studies have identified many potential biomarkers and pathways related to ovarian cancer, which increase understanding of ovarian cancer pathogenesis and could facilitate clinical diagnosis^10^,^11^,^12^,^13^,^14^. However, few studies have investigated metabolic alterations associated with responses to EOC treatment and disease relapse.

In this study, four batches of plasma samples were collected from the following patients: 35 pairs of EOC patients at the time of diagnosis (primary EOC patients) and after cytoreductive surgery (post-operative EOC patients), 35 matched controls, and 35 matched recurrent EOC patients. Metabolic profiling of these samples was performed by rapid resolution liquid chromatography mass spectrometry (RRLC/MS). First of all, potential biomarkers were identified for the primary, surgical and recurrent EOC, respectively. Then, metabolic changes of primary EOC biomarkers were monitored in the post-operative and recurrent EOC patients. Our ultimate goals were (1) to investigate metabolic alterations that underlie the responses to advanced EOC, surgical intervention and disease relapse; and (2) to discover potential biomarkers that could monitor EOC surgical efficacy and disease relapse.

## Results

### Enrolled patient characteristics

Thirty-five plasma samples from primary EOC patients, 35 from the same EOC patients after surgery, 35 from relapsed EOC patients, and 35 from controls were included in this study. The controls were age- and menopause-matched with primary EOC patients, and the recurrent EOC patients were age-, menopause- and stage-matched with primary EOC patients. According to the International Federation of Gynecology and Obstetrics (FIGO) staging system, 31 of the EOC patients were classed as stage III and four as stage IV. All EOC patients enrolled in this study had received cytoreductive surgery followed by platinum-based chemotherapy. There were no statistical differences observed for age, menopause, stage, weight, lymph node status, histology type, and histology differentiation among the four groups (*P* > 0.05). Detailed clinical characteristics for the participants are listed in Table 1.

### Metabolic profiles of primary EOC patients vs. matched controls

**Figure 1:**
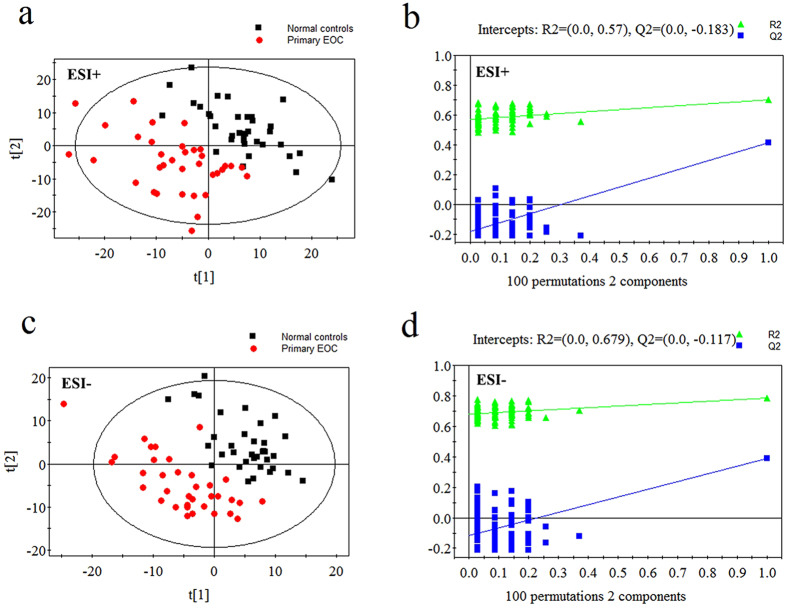
PLS-DA score plots and validation plots for primary EOC patients *versus* controls. (**a**) PLS-DA score plot in ESI+ mode (two components, R2 = 0.704, Q2 = 0.412); (**b**) validation plot in ESI+ mode; (**c**) PLS-DA score plot in ESI− mode (two components, R2 = 0.785, Q2 = 0.388); (**d**) validation plot in ESI− mode.

Typical chromatograms of our plasma samples are shown in Supplementary Fig. S1. The final data set consisted of 1924 ions (peaks) in ESI+ mode and 1428 in ESI- mode. The PCA performed on all the samples revealed that the QC samples were tightly clustered in PCA score plots, indicating the robustness of our metabolic profiling platform^15^. Because of the high complexity and variance in the data, the unsupervised method PCA could not detect obvious separations among the four groups (see Supplementary Fig. S2). The supervised method PLS-DA was performed, and prediction models were established. As shown in the PLS-DA score plots, the plasma samples from primary EOC patients and well-matched controls were clearly separated into two categories (Fig. 1a,c). The validation plots from permutation tests strongly supported the validity of the established PLS-DA models(Fig. 1b,d). Validity was supported by the result that all permuted R2 and Q2 values on the left were lower than the original point on the right, and that the Q2 regression line in blue had a negative intercept^16^. With the criteria of VIP >1 and *P* < 0.05, 37 metabolites were identified as potential biomarkers for primary EOC. Detailed information of metabolite identification can be referred to Supplementary Figs S3–S8. In the primary EOC patients, these markers included elevated l-kynurenine, hypoxanthine, and hydroxyphenyllactic acid, and decreased lysophosphatidylcholines (LPCs), fatty acids, amino acids, and bile acids. The metabolites, including biochemical characteristics, statistical information and metabolic pathways, are detailed in Supplementary Table S1.

### Metabolic profiles of pre- and post-operative EOC patients

**Figure 2:**
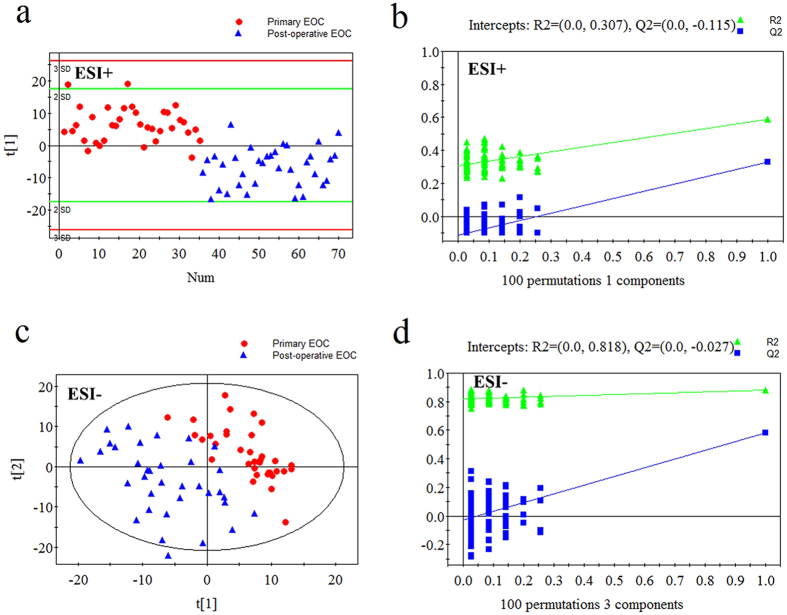
PLS-DA score plots and validation plots for primary EOC patients *versus* post-operative EOC patients. (**a**) PLS-DA score plot in ESI+ mode (one components, R2 = 0.588, Q2 = 0.327); (**b**) validation plot in ESI+ mode; (**c**) PLS-DA score plot in ESI− mode (three components, R2 = 0.881, Q2 = 0.584); (**d**) validation plot in ESI− mode.

The PLS-DA score plots revealed excellent classifications between primary and post-operative EOC patients (Fig. 2a,c). The validation tests supported the validity of the PLS-DA models (Fig. 2b,d). Altogether, 30 metabolites were important in the sample classification, as indicated by VIP >1 and *P* < 0.05. Post-operative EOC patients were characterized by increased fatty acids, amino acids, and uric acid, and decreased LPCs, l-kynurenine, and indoxylsulfuric acid (see Supplementary Table S2).

### Metabolic profiles of primary EOC patients vs. recurrent EOC patients, and post-operative EOC patients vs. recurrent EOC patients

**Figure 3:**
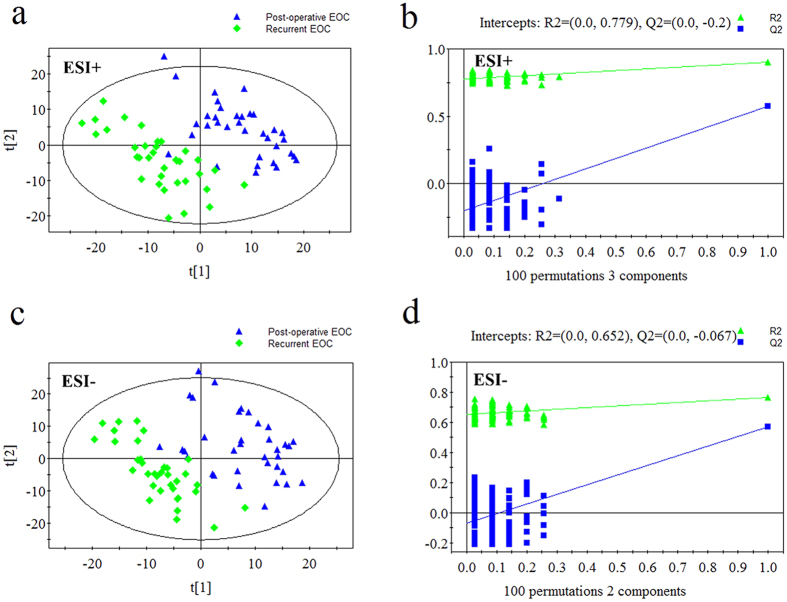
PLS-DA score plots and validation plots for post-operative EOC patients *versus* recurrent EOC patients. (**a**) PLS-DA score plot in ESI+ mode (three components, R2 = 0.899, Q2 = 0.575); (**b**) validation plot in ESI+ mode; (**c**) PLS-DA score plot in ESI− mode (two components, R2 = 0.765, Q2 = 0.572); (**d**) validation plot in ESI− mode.

**Figure 4:**
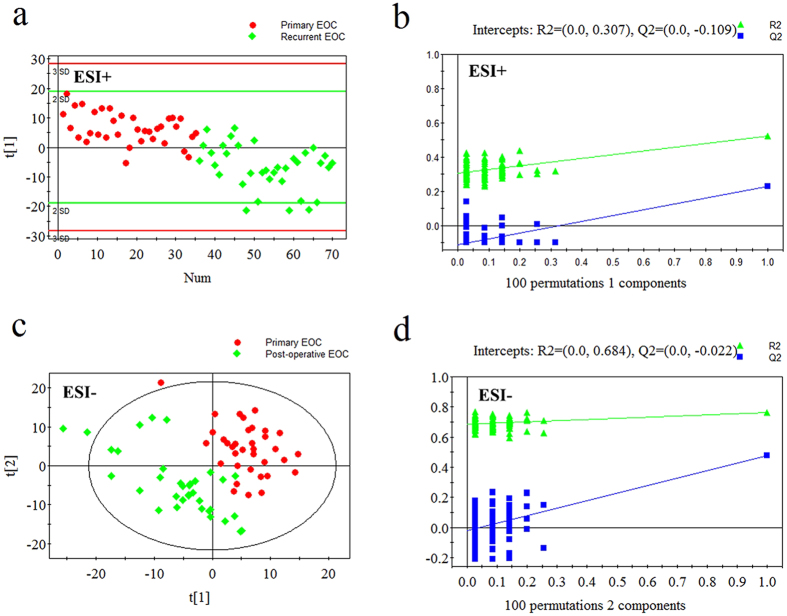
PLS-DA score plots and validation plots for primary EOC patients *versus* recurrent EOC patients. (**a**) PLS-DA score plot in ESI+ mode (one component, R2 = 0.522, Q2 = 0.230); (**b**) validation plot in ESI+ mode; (**c**) PLS-DA score plot in ESI− mode (two components, R2 = 0.764, Q2 = 0.475); (**d**) validation plot in ESI− mode.

The PLS-DA score plots also showed differences between post-operative EOC patients and recurrent EOC patients, and between primary and recurrent EOC patients (Figs 3 and 4). Using the same criteria of VIP >1 and *P* < 0.05, 42 differential metabolites between post-operative and recurrent EOC patients and 26 between primary and recurrent EOC patients were selected. Compared with post-operative EOC patients, recurrent EOC patients showed increased LPCs, lysophosphatidylethanolamines (LPEs) and amino acids, and decreased 2-octenoic acid and creatine (see Supplementary Table S3). Compared with primary EOC patients, the levels of a series of lipids, amino acids and their related metabolites were remarkably increased in recurrent EOC patients (see Supplementary Table S4).

### Dynamic changes of 37 primary EOC biomarkers across advanced EOC, surgery and recurrence

**Figure 5:**
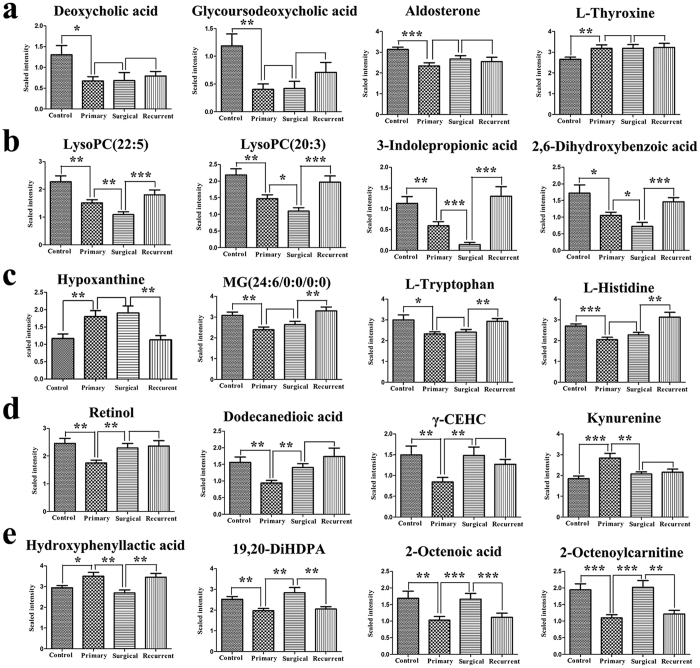
Error bar chart (Mean + Standard error) for demonstrating dynamic changes of primary EOC biomarkers across advanced EOC, surgery and recurrence (*P < 0.05, **P < 0.01, ***P < 0.001, P values bigger than 0.05 are not marked).

Metabolite levels of 37 primary EOC biomarkers were investigated further in the post-operative EOC patients and recurrent EOC patients. Multiple patterns of change in the levels of these biomarkers were observed. Some metabolites, including aldosterone and deoxycholic acid, remained unchanged in the groups of primary, post-operative and recurrent EOC patients (Fig. 5a). Some metabolites (e.g. LPC(22:5) and 3-indolepropionic acid) showed the same directional trends in both primary and post-operative EOC patients, but tended to recover towards levels of the control group in the recurrent EOC patients (Fig. 5b). Some metabolites, such as l-tryptophan and L-histidine, were unchanged from pre- to post-operative but changed from post-operative to recurrent EOC patients (Fig. 5c). Most remarkably, eight metabolites in the post-operative EOC patients, including l-kynurenine, γ-CEHC, 2-octenoylcarnitine, retinol, 2-octenoic acid, dodecanedioic acid, 19,20-DiHDPA and hydroxyphenyllactic acid, showed recovery towards levels of the control group (Fig. 5d). The levels of four of these metabolites (2-octenoylcarnitine, 2-octenoic acid, 19,20-DiHDPA and hydroxyphenyllactic acid) went back again to primary EOC levels in recurrent EOC patients (Fig. 5e). Among the metabolites discussed, these eight metabolites show the most potential for monitoring surgical efficacy and/or disease relapse. These changes for all metabolites are detailed in Supplementary Table S5.

## Discussion

We investigated specific metabolic signatures for advanced EOC at diagnosis, surgery and relapse. Potential biomarkers were identified for primary, post-operative and recurrent EOC patients. Some metabolites exhibited excellent potential for monitoring EOC patients.

Recent studies have demonstrated that plasma metabolic profiles in women are age and menopause dependent^17^,^18^,^19^. Consequently, it is crucial to control these two factors in metabolomic studies to avoid false discoveries. In this study, these two factors were well controlled to allow for identification of specific biomarkers related to EOC. In line with previous reports of metabolomic studies on ovarian cancer, primary EOC patients exhibited altered metabolism of phospholipids, l-tryptophan and l-histidine, and piperidine derivatives^12^,^13^. Identification of some novel metabolite markers in this study probably resulted from the use of a different research design and improved capacity of metabolite identification compared with earlier studies. Compared with controls, significantly lower concentrations of tetracosahexaenoic acid, 2-octenoic acid, 12,13-DiHODE and 19,20-DiHDPA were observed in primary EOC patients. Knowledge of changes in these lipid-related metabolites supplements earlier understanding of lipid metabolism in EOC and further supports the finding that lipid metabolism is significantly disturbed in EOC^20^. It has been suggested that changes in lipid metabolism can affect numerous cellular processes, including cell growth, proliferation, differentiation and motility, which in turn contributes to tumourigenesis and malignancy^21^. *p*-Salicylic acid, a metabolite involved in ubiquinone biosynthesis, decreased in primary EOC patients. This could be an indication of energy disorders commonly seen in EOC, as ubiquinone is known to play an important role in mitochondrial respiratory and energy production^22^. Energy disorders are also indicated by the elevated hypoxanthine in primary EOC patients, as hypoxanthine is a potential generator of oxygen radicals and sensitive indicator of hypoxia^23^.

Therapeutic surgery for EOC aims to remove as much of the tumor as possible^24^. Metabolic changes between pre- and post-operative EOC patients could be attributed to oxidative stress, nutritional supplementation, or surgical curative effect^25^. Down-regulations of 3-indolepropionic acid and indoxylsulfuric acid might be indicative of altered oxidative stress in post-operative EOC patients, as both these metabolites have been suggested to be involved in oxidative stress^26^,^27^. This is further supported by the decreased uric acid levels seen in post-operative EOC patients, because uric acid has an important role as an oxidative stress marker^28^. Many significantly elevated metabolites in the post-operative EOC patients (e.g. capric acid, caprylic acid, and α-linolenic acid) may be attributable to nutritional supplementation (e.g. fat emulsion for intravenous injection during surgery), which is routinely performed to improve clinical outcome in major abdominal surgery^29^. Remarkably, we observed that the plasma levels of eight metabolites in the post-operative EOC patients trended towards recovering to the levels of the controls. This recovery is probably in response to the tumor removal. Therefore, these eight metabolites show great potential for monitoring EOC surgical efficacy.

Recurrent EOC patients are usually characterized by one of the biggest mysteries in cancer research, chemotherapy resistance, which accounts for most therapeutic failures and eventual death^30^. Compared with primary EOC patients, relapsed EOC patients showed substantial metabolic alterations. A series of amino acids (e.g. l-histidine, l-tryptophan, and l-phenylalanine) and amino acid-related metabolites (kynurenine, 2,3-dihydroxyvaleric acid, glyceric acid, and α-ketoisovaleric acid) were remarkably increased in relapsed EOC patients compared with the primary EOC patients. Further significant alterations in recurrent EOC patients were observed within lipid metabolism, as indicated by significantly increased levels of LPCs, LPEs and fatty acids. Altered pathways of amino acids and lipids have been suggested to contribute to platinum resistance in ovarian cancer cells^31^. Recent studies have also suggested that adverse lipid profile raises prostate cancer recurrence risk^32^. Therefore, alterations of these metabolites might serve as specific biomarkers for EOC recurrence and possibly as metabolism-based drug targets. Notably, the enhanced conversion of l-tryptophan to l-kynurenine by indoleamine-pyrrole 2,3-dioxygenase (IDO) has been well characterized in ovarian cancer^33^,^34^, and was evident in this study as well. Multiple clinical trials are currently evaluating IDO inhibitors for cancer immunotherapy^34^. Nevertheless, this reaction does not appear to be important in recurrent EOC patients, because l-kynurenine did not recover towards primary EOC levels in recurrent EOC patients. Therefore, our findings suggest that while IDO inhibitors may be promising for first-line therapy, they may not be applicable to resistant ovarian cancer.

Importantly, the levels of 2-octenoylcarnitine, 2-octenoic acid, 19,20-DiHDPA and hydroxyphenyllactic acid in the post-operative EOC patients trended towards recovering to levels of the control group but then trended towards primary EOC levels after disease relapse. As discussed above, recovery of these metabolites in the post-operative EOC patients could be attributed to surgical efficacy. Their recurrence in relapsed EOC patients is most likely caused by resurgent tumor proliferation. These four biomarkers are excellent potential biomarkers for monitoring EOC progression.

To the best of our knowledge, this study is the first to examine plasma metabolic changes in response to EOC surgery and recurrence. However, there were several limitations. One is the relatively small sample size in each group, which might prevent the differences in some metabolites from being fully apparent. In addition, a targeted and quantitative analysis of the potential biomarkers is needed to advance clinical translation. For future research, we are collecting a larger sample cohort and performing a targeted metabolomic investigation into these biomarkers.

In summary, we present a holistic view of the plasma metabolic changes related to advanced EOC at diagnosis, surgery and relapse. Specific metabolite markers have been identified that could be used to monitor surgery efficacy and disease relapse. Primary EOC is characterized by abnormal lipid metabolism, energy disorders and altered l-tryptophan metabolism. The phenomena of oxidative stress and surgical efficacy are clear in the metabolic profiles of post-operative EOC patients. Metabolic signatures in recurrent EOC patients show increased amino acid and lipid metabolism compared with primary EOC patients. In all, this study highlights a number of potential EOC biomarkers that could facilitate both understanding and monitoring of advanced EOC, its remission and progression.

## Methods

### Plasma samples

The present experiments were conducted in accordance with the 1975 Declaration of Helsinki. The protocol in this study was approved by the Ethics Committee of the Tumor Hospital at Harbin Medical University, Harbin, China. Plasma samples were collected by the Department of Gynecology of the Tumor Hospital at Harbin Medical University between January 2012 and July 2013. All the participants gave informed written consent. The inclusion criteria and exclusion criteria were as follows: (1) participants suffering from metabolic, liver, or kidney diseases or any other cancers were excluded; (2) controls were defined as uterine fibroid patients or benign ovarian tumor patients; (3) primary EOC patients and controls were not taking any medications, and their diagnosis was confirmed by histopathology after surgery; (4) blood samples from post-operative EOC patients were collected within 3–7 days of cytoreductive surgery; (5) recurrent EOC patients were confirmed by follow-up blood tests, physical examinations, and imaging tests. After fasting for 12 h, 5 mL of whole blood was collected from each patient into an EDTA tube. Fresh blood was centrifuged at 1,000 ×*g* for 10 min, and the supernatant was collected and frozen at −80 °C until analysis.

### Quality control (QC) samples

To ensure the stability and repeatability of the RRLC/MS systems, QC samples were used in both electrospray ionization positive (ESI+) and negative (ESI−) modes. Pooled QC samples were prepared from a mixture of all plasma samples. One QC sample was run after every twelve samples.

### Sample pretreatment

In order to ensure a balance in sample size across groups over time, sample pretreatment and RRLC/MS analysis were performed using the blocked randomization method^35^. The block size was set at 4, so there are 24 possible ways to equally assign samples to a block. Allocation proceeds by randomly selecting one of the orderings and assigning the samples according to the specified sequence. After the plasma samples were thawed in a 4 °C refrigerator, 200 μL of plasma was mixed with five times the volume (1000 μL) of acetonitrile at 4 °C. The samples were centrifuged at 4,000 ×*g* for 10 min at 4 °C. The supernatant was transferred into a clean vial, and evaporated until dry, under reduced pressure. The residue was dissolved in 100 μLof acetonitrile/water (3:1, *v*/*v*), vortex-mixed for 1 min, and then centrifuged at 12,000 ×*g* for 15 min at 4 °C. The supernatant was then placed into the sample vial for analysis.

### Chromatography

A 10 μL aliquot of the pre-treated sample was injected into a 3.0 × 100 mm (1.8 mm) ZORBAX SB-C18 column (Agilent Technologies, Santa Clara, CA, USA) for RRLC (1260 series; Agilent Technologies). The mobile phase for ESI+ was a mixture of acetonitrile containing 0.1% formic acid (A) and water containing 0.1% formic acid (B). The mobile phase for ESI− was a mixture of acetonitrile (A) and water (B). A linear mobile phase gradient was used as follows: 2% A, held for 1 min; 1–18 min, increased to 98% A; 18–21 min, held at 98% A; 21–21.1 min, decreased to 2%A; and 21.1–28 min, held at 2% A. The mobile phase flow rate was 0.3 mL/min at 40 °C.

### Mass spectrometry

Mass spectrometry was performed with an Agilent 6530-QTOF (Agilent Technologies) operating in both ESI+ and ESI− modes. The capillary voltage was set as 4.0 kV for ESI+ mode and 3.5 kV for ESI− mode. Nitrogen was used as the desolvation gas at a flow rate of 10 L/min. The desolvation temperature was 350 °C. Centroid data were collected in full scan mode from 50 to 1000 *m*/*z*.

### Data preprocessing and annotation

The XCMS package was used to preprocess the raw data in the R platform, including peak detection, peak matching, matched filtration, and retention time alignment^36^. The default XCMS parameters were used, with the following exceptions: xcmsSet (method = “centWave”, peakwidth = c(10,60)); group (bw = 20, minfrac = 0.6); retcor (method = “obiwarp”). The preprocessed results generated a data matrix that consisted of the retention times, mass-to-charge ratio (*m*/*z*) values, and peak intensities. CAMERA in R was then used to annotate isotope peaks, adducts and fragments in the peak lists^37^. Isotopic peaks were excluded before statistical analysis.

### Statistical analysis

The preprocessed data was exported to SIMCA-P 11.5 software for multivariate analysis. Principal component analysis (PCA) was first used to detect separation trends and outliers^38^. Partial least-squares discriminant analysis (PLS-DA) was then performed to understand global metabolic changes between groups. To avoid overfitting, permutation tests with 100 iterations were performed to validate the supervised model^39^. Variable importance in the projection (VIP) for each metabolite was calculated based on the established PLS-DA model. In addition, Welch’s *t*-test was used to further validate the significance of each metabolite. Potential metabolic biomarkers were selected using VIP > 1 and *P* < 0.05. To visualize metabolite changes across advanced EOC, surgery and recurrence, the standardized metabolite level in the four groups was plotted in an error bar chart.

## Additional Information

**How to cite this article**: Ke, C. *et al*. Metabolic phenotyping for monitoring ovarian cancer patients. *Sci. Rep*. **6**, 23334; doi: 10.1038/srep23334 (2016).

## Supplementary Material

Supplementary Information

## Figures and Tables

**Table 1:** Clinical characteristics of the participants enrolled in this study.

Characteristics	Controls (n = 35)	Primary EOC (n = 35)	Post-operative EOC (n = 35)	Relapsed EOC (n = 35)	P value†
Age (mean ± SD*)	53.22 ± 8.30	53.67 ± 9.04	53.76 ± 9.05	54.31 ± 7.28	0.9616^a^
Weight (median/range)	60 (45 ~ 87)	60 (50 ~ 91.5)		63.50 (44 ~ 88)	0.1359^b^
Menopause (pre/post)	11/24	11/24		11/24	1.0000^c^
CA125(median/range)	17.45 (5.06 ~ 184.5)	490.80 (67.23 ~ 5000.00)‡		95.10 (21.06 ~ 2048.00)	<0.0001^b^
FIGO Stage (III/IV)	–	31/4		31/4	1.0000^c^
Lymph node metastasis (Yes/No)	–	20/15		17/18	0.4726^c^
Histology differentiation					0.7936^c^
Well	–	5		4	
Moderately	–	11		14	
Poorly	–	19		17	
Histology type					0.7022^c^
Serous	–	27		25	
Mucoid	–	3		2	
Endometrioid	–	5		8	

^*^SD: standard deviation.

^†^P value refers to the statistical significance of differences among or between groups: ^a^One-way analysis of variance was performed. ^b^Nonparametric Kruskal-Wallis rank sum test was performed. ^c^Chi-square (χ^2^) test or Fisher’s exact test was performed.

^‡^The CA125 value is measured as the pre-operative value.
