# Supplementary material for: Metabolic phenotyping for monitoring ovarian cancer patients
Source: Sci Rep. 2016 Mar 21;6:23334. doi: 10.1038/srep23334 (PMC4800393; doi:10.1038/srep23334)
Supplement: Supplementary Information [file srep23334-s1.pdf]

Supplementary materials

Metabolic phenotyping for monitoring ovarian cancer patients

Chaofu Ke[a], Ang Li[a], Yan Hou[a], Meng Sun[b], Kai Yang[a], Jinlong Cheng[c], Jingtao Wang[a], Tingting Ge[c], Fan Zhang[a], Qiang Li[a], Junnan Li[a], Ying Wu[a], Ge Lou[c] and Kang Li[a].

[a] Department of Epidemiology and Biostatistics, School of Public Health, Harbin Medical University, Harbin 150086, P.R. China.

[b] Department of Cardiology, The Second Affiliated Hospital of Harbin Medical University, and The Key Laboratory of Myocardial Ischemia, Harbin Medical University, Harbin 150086, P.R. China.

[c] Department of Gynecology Oncology, The Tumor Hospital, Harbin Medical University, Harbin 150086, P. R. China.

Address correspondence to:

Kang Li, Ph.D., Department of Epidemiology and Biostatistics, School of Public Health, Harbin Medical University, Harbin 150086, P.R. China. E-mail: likang@ems.hrbmu.edu.cn; and Ge Lou, M.D., Department of Gynecology Oncology, The Tumor Hospital, Harbin Medical University, Harbin 150086, P.R. China. E-mail: louge@ems.hrbmu.edu.cn

Strategy of metabolite identification

First, peak lists and annotation results obtained by R package XCMS[1] and CAMERA[2] were analyzed to find quasi-molecular ions and determine the corresponding molecular weights.

Second, the exact molecular weights of the monoisotopic ions were used to search the online databases, such as HMDB[3] (http://www.hmdb.ca/), METLIN[4] (http://metlin.scripps.edu/) and MassBank[5] (http://www.massbank.jp/). The mass tolerance between the measured m/z values and the exact mass of the components of interest was set within 15 ppm.

Third, a QTOF mass analyzer (6530, Agilent Technologies) was used to produce fragmentation patterns and structure information for biomarker candidates in the MS/MS experiments. The MS/MS fragmentation patterns of the biomarkers were compared to the spectral data of metabolites that had the same m/z in HMDB, METLIN or MassBank.

Finally, if available, confirmation with standards was performed by comparison of retention time, isotopic distribution, and fragments of reference standards (Sigma-Aldrich) with those obtained in real samples.

Metabolite identification was classed into 4 levels based on the Metabolomics Standard Initiative[6]:

The first level (level 1): Metabolites are verified by reference standards. Fig. S3 and Fig. S4 are representative identification procedures using reference standards.

The second level (level 2): Metabolites are identified based on accurate mass data, retention time, experimental MS/MS spectra, and library MS/MS spectra. Fig. S5 and Fig. S6 are representative identification procedures based on MS/MS spectral matching.

The third level (level 3): Metabolites are identified based on accurate mass data, retention time, experimental MS/MS spectra, and knowledge of characteristic MS/MS ions spectra. Fig. S7 and Fig. S8 are representative identification procedures based on characteristic MS/MS ions spectra.

The fourth level (level 4): Metabolites are speculated based on accurate mass data, retention time, and biological relevance. Metabolites in this level would be further validated in the future with the improvement of library spectra and availability of reference standards.

Reference:

1 Smith, C. A., Want, E. J., O'Maille, G., Abagyan, R. & Siuzdak, G. XCMS: processing mass spectrometry data for metabolite profiling using nonlinear peak alignment, matching, and identification. *Anal Chem* **78**, 779-787, (2006).

2 Kuhl, C., Tautenhahn, R., Bottcher, C., Larson, T. R. & Neumann, S. CAMERA: an integrated strategy for compound spectra extraction and annotation of liquid chromatography/mass spectrometry data sets. *Anal Chem* **84**, 283-289, (2012).

3 Wishart, D. S. *et al.* HMDB 3.0--The Human Metabolome Database in 2013.

Nucleic acids research **41**, D801-807, (2013).

4 Smith, C. A. *et al.* METLIN: a metabolite mass spectral database. *Ther Drug Monit* **27**, 747-751, (2005).

5 Horai, H. *et al.* MassBank: a public repository for sharing mass spectral data for life sciences. *J Mass Spectrom* **45**, 703-714, (2010).

6 Sumner, L. W. *et al.* Proposed minimum reporting standards for chemical analysis Chemical Analysis Working Group (CAWG) Metabolomics Standards Initiative (MSI). *Metabolomics* **3**, 211-221, (2007).

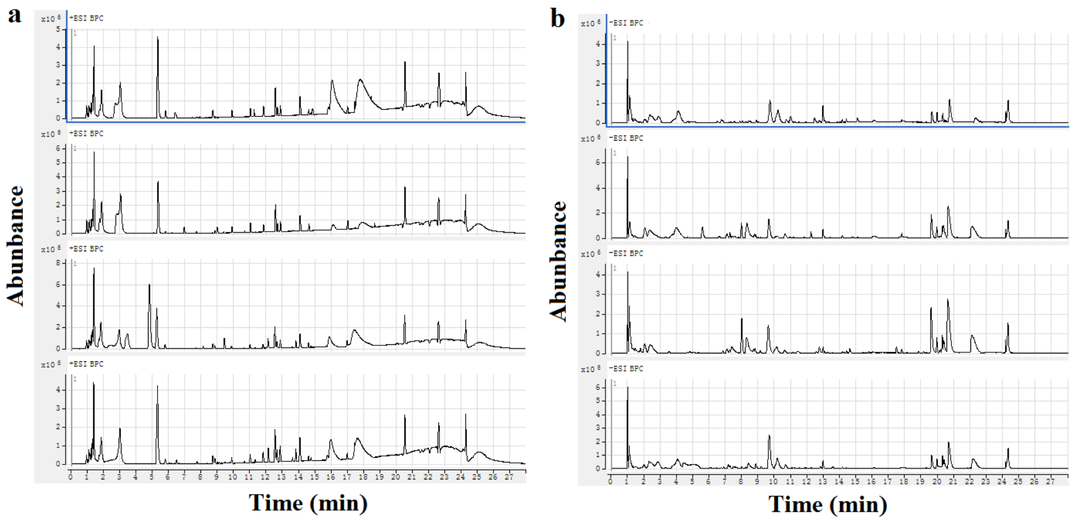

Fig. S1 Typical RPLC-QTOF/MS chromatograms of plasma samples for a control woman, a primary EOC patient, a post-operative EOC patient and a recurrent EOC patient in ESI+ mode (a) and ESI- mode (b), respectively.

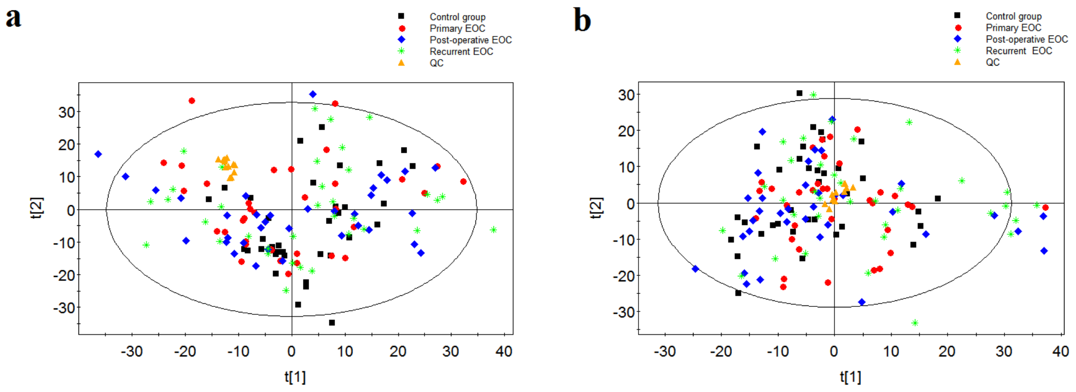

Fig. S2 PCA score plots for discriminating control samples, primary EOC samples, post-operative EOC samples, relapsed EOC samples and QC samples in ESI+ mode (a) and ESI- mode (b), respectively.

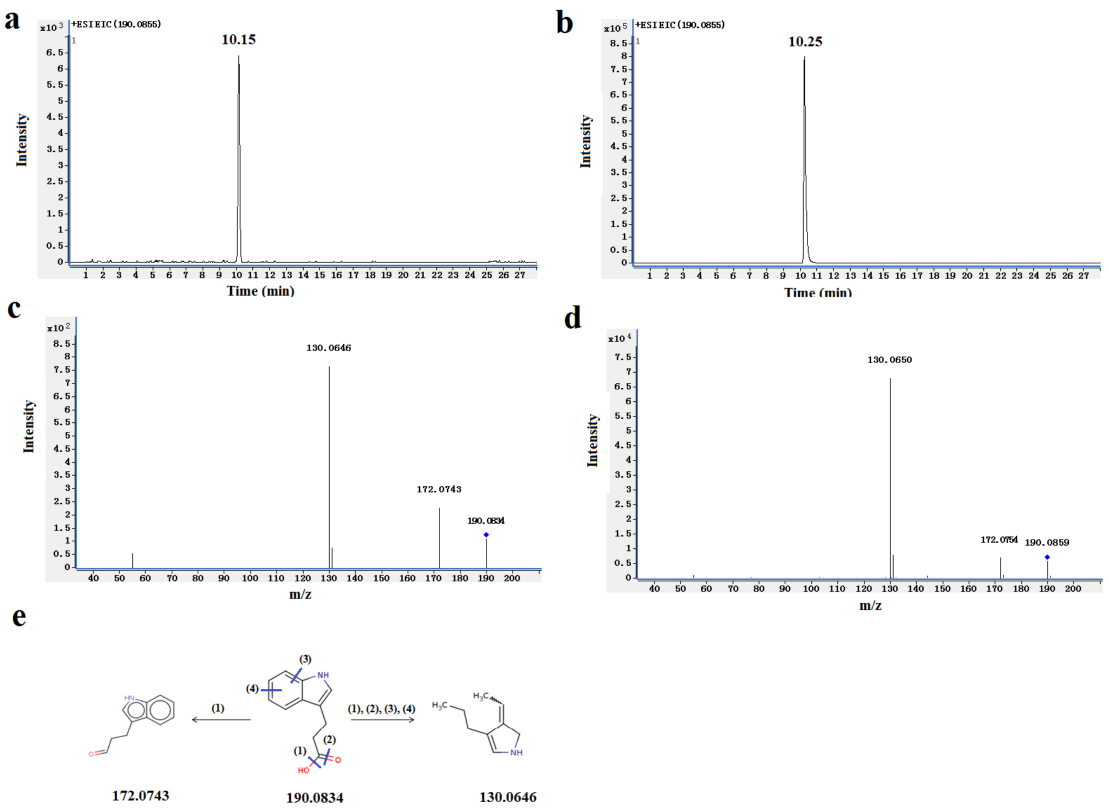

Fig. S3 The identification information of potential biomarker 3-indolepropionic acid in ESI+ mode. (a) The extracted ion chromatographic peak of quasi-molecular ion ([M+H]$^+$) at m/z 190.0855 in a plasma sample. (b) The extracted ion chromatographic peak of quasi-molecular ion ([M+H]$^+$) of reference standard 3-indolepropionic acid. (c) The MS/MS spectrum of quasi-molecular ion ([M+H]$^+$) at m/z 190.0855 at 10.15 min in a plasma sample. (d) The MS/MS spectrum of quasi-molecular ion ([M+H]$^+$) of reference standard 3-indolepropionic acid. (e) The fragment ions of 3-Indolepropionic acid.

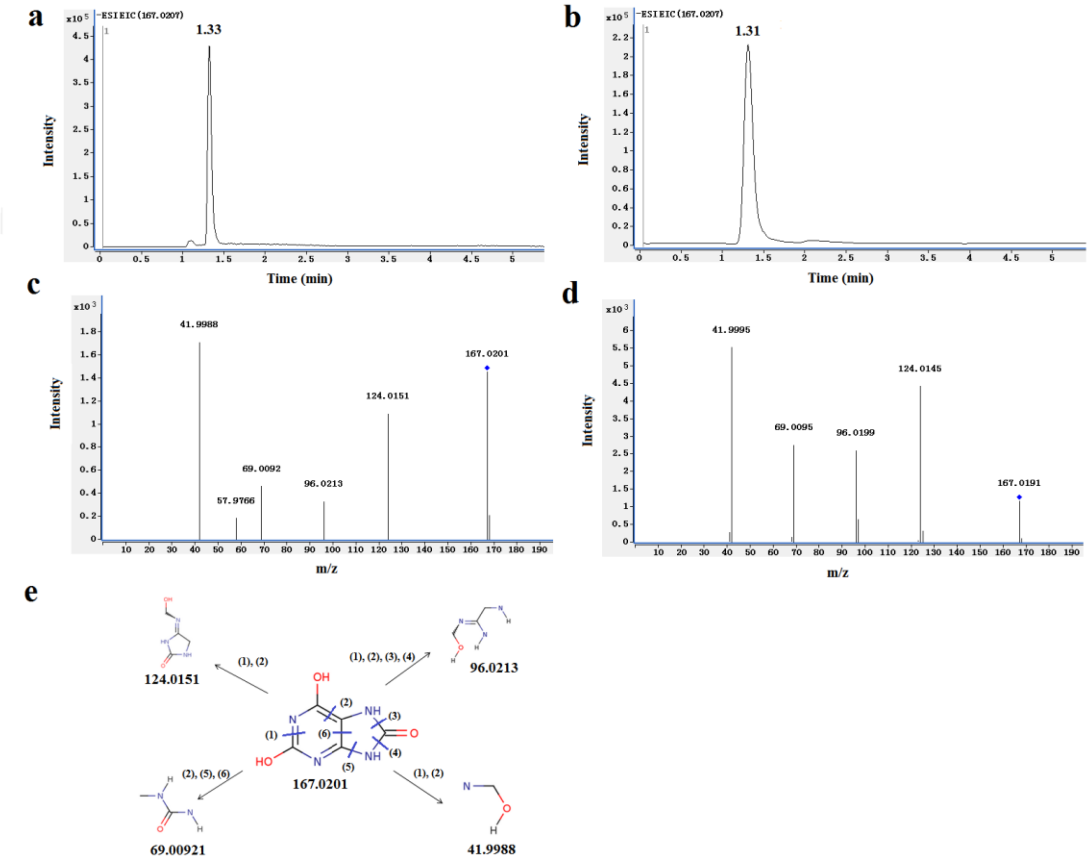

Fig. S4 The identification information of potential biomarker uric acid in ESI- mode. (a) The extracted ion chromatographic peak of quasi-molecular ion ($[M\text{-}H]^-$) at m/z 167.0207 in a plasma sample. (b) The extracted ion chromatographic peak of quasi-molecular ion ($[M\text{-}H]^-$) of reference standard uric acid. (c) The MS/MS spectrum of quasi-molecular ion ($[M\text{-}H]^-$) at m/z 167.0207 at 1.33 min in a plasma sample. (d) The MS/MS spectrum of quasi-molecular ion ($[M\text{-}H]^-$) of reference standard uric acid. (e) The fragment ions of uric acid.

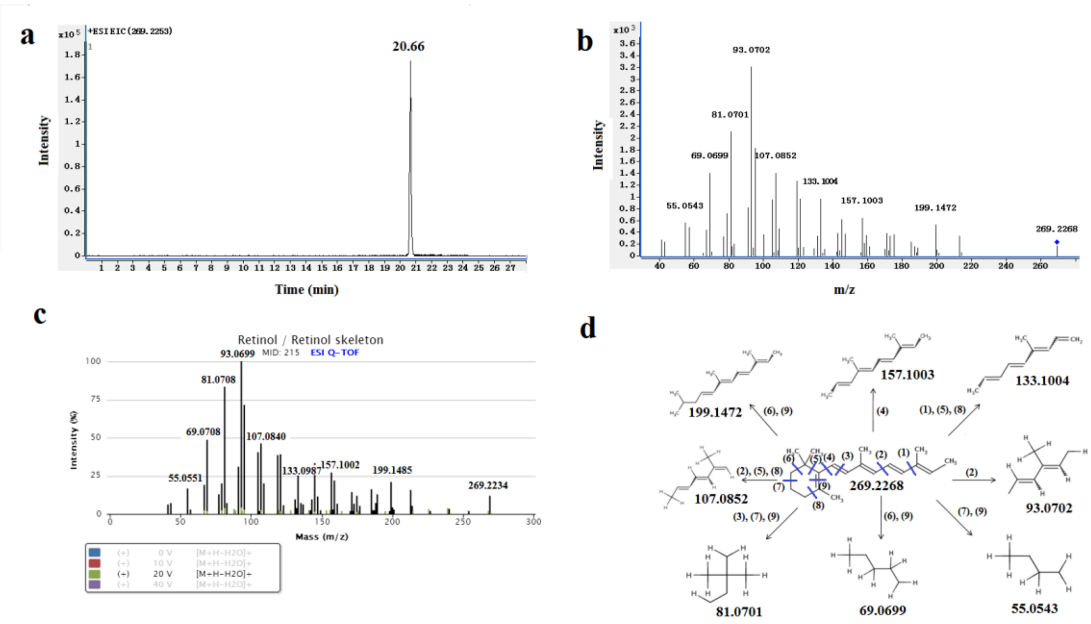

Fig. S5 The identification information of potential biomarker retinol in ESI+ mode. (a) The extracted ion chromatographic peak of quasi-molecular ion ($[M+H\text{-}H2O]^+$) at m/z 269.2253 in a plasma sample. (b) The MS/MS spectrum of quasi-molecular ion ($[M+H\text{-}H2O]^+$) at m/z 269.2253 at 20.66 min in a plasma sample. (c) The MS/MS spectrum of quasi-molecular ion ($[M+H\text{-}H2O]^+$) of retinol in the METLIN database. (d) The fragment ions of retinol.

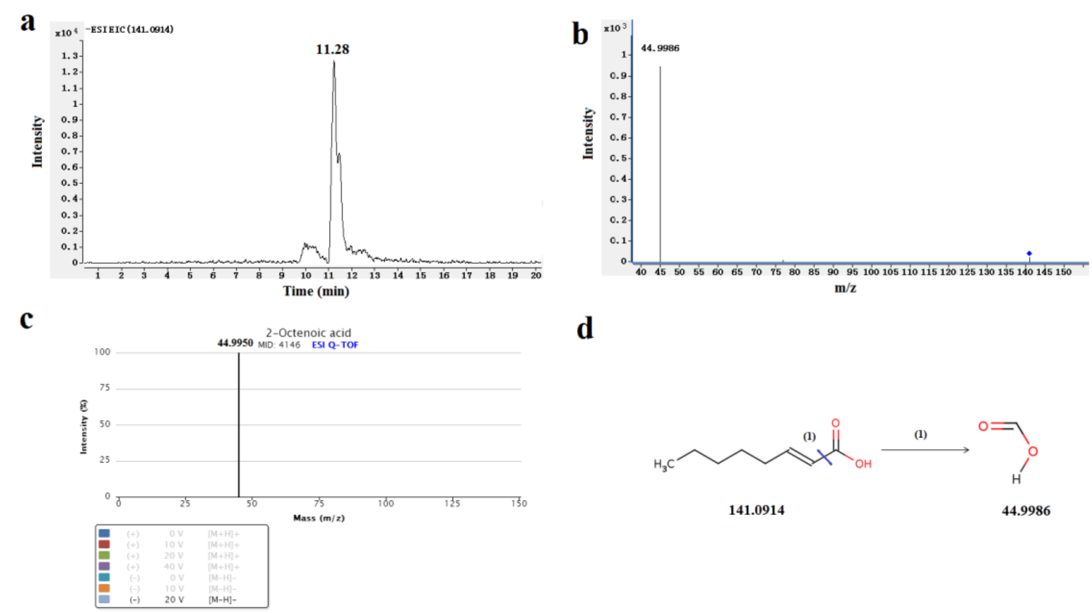

Fig. S6 The identification information of potential biomarker 2-octenoic acid in ESI- mode. (a) The extracted ion chromatographic peak of quasi-molecular ion ($[M\text{-}H]^-$) at m/z 141.0914 in a plasma sample. (b) The MS/MS spectrum of quasi-molecular ion ($[M\text{-}H]^-$) at m/z 141.0914 at 11.28 min in a plasma sample. (c) The MS/MS spectrum of quasi-molecular ion ($[M\text{-}H]^-$) of 2-Octenoic acid in the METLIN database. (d) The fragment ions of 2-Octenoic acid.

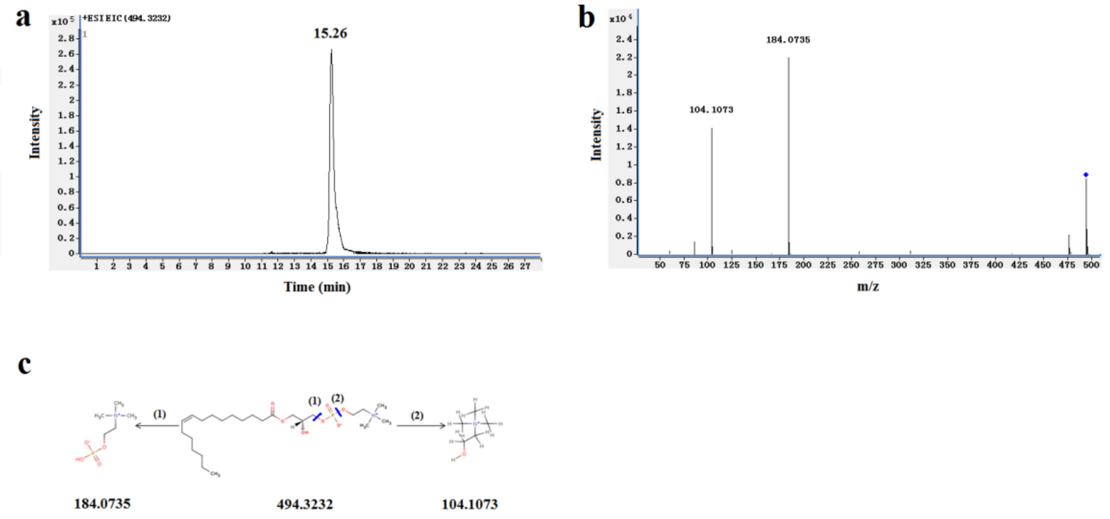

Fig. S7 The identification information of potential biomarker LysoPC(16:1) in ESI+ mode. (a) The extracted ion chromatographic peak of quasi-molecular ion ($[M+H]^+$) at m/z 494.3232 in a plasma sample. (b) The MS/MS spectrum of quasi-molecular ion ($[M+H]^+$) at m/z 494.3232 at 15.26 min in a plasma sample. (c) The characteristic fragment ions (184.0735, 104.1073) of LysoPC(16:1).

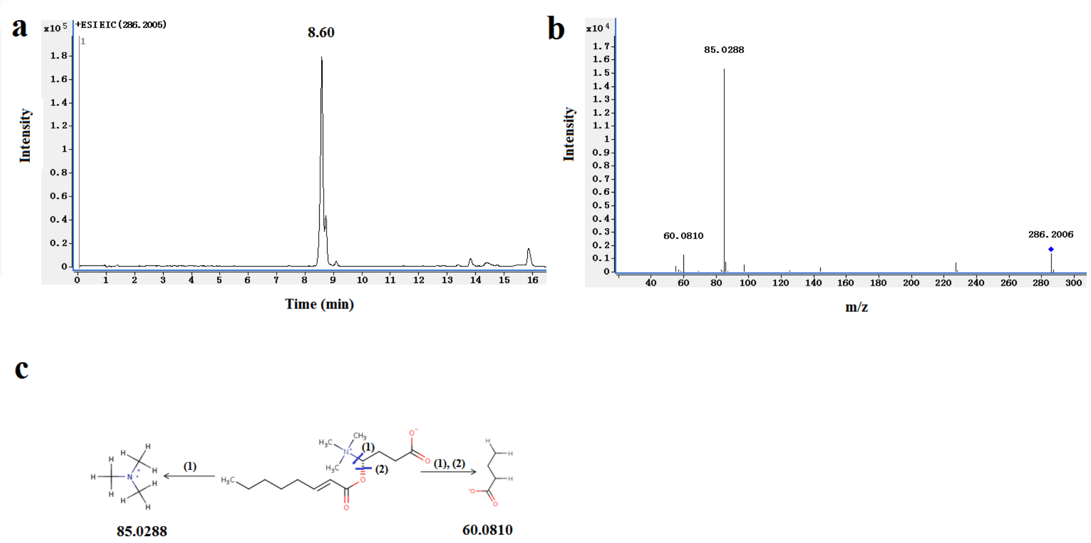

Fig. S8 The identification information of potential biomarker 2-octenoylcarnitine in ESI- mode. (a) The extracted ion chromatographic peak of quasi-molecular ion ($[M+H]^+$) at m/z 286.2005 in a plasma sample. (b) The MS/MS spectrum of quasi-molecular ion ($[M+H]^+$) at m/z 286.2005 at 8.60 min in a plasma sample. (c) The characteristic fragment ions (85.0288, 60.0810) of 2-octenoylcarnitine.

Table S1 The detailed information about 37 potential biomarkers that are differential between primary EOC patients and controls

No.	Metabolite	m/z	RT(min)	ppm	CV%	L[a]	FC[b]	P	VIP	Pathway
Positive-ion electrospray ionization mode (ESI+)										
1	LysoPC(18:2)	520.3366	16.04	6.14	2.69	3	-0.60	3.20E-06	2.69	Glycerophospholipid metabolism
2	LysoPC(18:3)	518.3227	14.76	2.70	4.16	3	-0.83	0.00018	2.27	Glycerophospholipid metabolism
3	LysoPC(14:0)	468.3073	14.61	2.56	4.54	3	-0.85	0.00019	2.24	Glycerophospholipid metabolism
4	LysoPC(18:1)	522.3535	18.60	3.64	2.20	3	-0.44	0.001012	2.05	Glycerophospholipid metabolism
5	LysoPC(16:1)	494.3232	15.26	1.82	4.26	3	-0.6	0.000806	2.06	Glycerophospholipid metabolism
6	LysoPC(22:5)	570.3534	16.53	3.51	3.16	3	-0.57	0.002168	1.94	Glycerophospholipid metabolism
7	LysoPC(20:3)	546.3544	17.07	1.83	3.27	3	-0.58	0.002075	1.93	Glycerophospholipid metabolism
8	LysoPC(20:4)	544.3369	15.83	5.33	3.16	3	-0.43	0.000748	2.05	Glycerophospholipid metabolism
9	LysoPE(18:2/0:0)	478.293	14.86	0.42	3.76	3	-0.52	0.002168	1.91	Lysophospholipid metabolism
10	LysoPE(0:0/18:1)	480.3082	16.16	0.62	1.90	3	-0.37	0.008377	1.63	Lysophospholipid metabolism
11	L-Tryptophan	205.0957	5.53	7.31	9.65	1	-0.32	0.011869	1.56	Tryptophan metabolism
12	Kynurenine	209.0914	3.35	3.35	11.19	1	0.59	0.00039	2.61	Tryptophan metabolism
13	3-Indolepropionic acid	190.0855	10.28	4.21	5.73	1	-1.47	0.005872	1.90	Tryptophan metabolism
14	Tetracosahexaenoic acid	357.2776	14.80	3.36	9.68	3	-1.86	0.003231	1.93	Linoleic acid metabolism
15	Aldosterone	361.1998	10.30	3.32	4.75	4	-0.58	9.51E-05	2.51	Steroidogenesis
16	Glycoursodeoxycholic acid	450.3201	13.02	2.89	5.82	4	-1.8	0.001835	2.30	Bile acid biosynthesis
17	Hypoxanthine	137.0460	1.43	1.46	2.41	1	0.85	0.004392	1.95	Purine metabolism
18	MG(24:6/0:0/0:0)	431.3139	16.63	3.94	4.66	4	-0.39	0.0014	1.93	Monoacylglyceride metabolism
19	2-Octenoylcarnitine	286.2005	8.89	2.74	3.91	3	-0.83	0.000109	2.46	Others
20	Piperine	286.1431	13.84	2.45	2.89	1	-2.37	0.015023	1.92	Lysine metabolism
21	2-Piperidinone	100.0757	4.30	0.00	11.54	1	-0.61	0.004895	1.79	Lysine metabolism
22	Retinol[c]	269.2253	20.44	5.94	6.53	2	-0.47	0.001617	1.95	Retinol metabolism
23	Dodecanedioic acid[d]	272.1850	7.83	2.20	6.19	4	-0.66	0.00119	2.03	Metabolism of lipids and carbohydrates
24	L-Thyroxine	777.6911	11.11	3.73	6.27	1	0.25	0.006899	2.09	Tyrosine metabolism
Negative-ion electrospray ionization mode (ESI-)										
25	12,13-DiHODE	311.2202	13.38	8.35	4.07	3	-0.74	0.000215	2.75	Fatty acid metabolism

26	2-Octenoic acid	141.0910	11.15	7.80	10.89	2	-0.65	0.008983	2.01	Fatty acid biosynthesis
27	19,20-DiHDPA	361.2359	14.61	6.92	6.87	3	-0.36	0.001648	2.71	Fatty acid metabolism
28	N-Undecanoylglycine	242.1750	11.05	4.96	3.17	4	0.32	0.021176	1.87	Fatty acid metabolism
29	γ-CEHC	263.1273	10.80	6.08	4.72	2	-0.95	0.008108	1.79	Vitamin E metabolism
30	p-Salicylic acid	137.0242	2.74	1.46	14.97	2	-1.1	0.002942	2.30	Ubiquinone biosynthesis
31	Nutriacholic acid	389.2665	12.91	8.22	8.76	4	-0.60	0.000338	2.48	Bile acid metabolism
32	Deoxycholic acid	391.2819	15.0133	8.94	5.15	1	-1.11	0.013663	1.80	Bile acid biosynthesis
33	Hydroxyphenyllactic acid	181.0497	1.46	4.97	2.32	2	0.22	0.013832	2.06	Tyrosine metabolism
34	4-Nitrophenol	138.0190	9.54	5.07	2.72	2	0.24	0.002721	2.29	Microbial metabolism
35	L-Histidine	154.0614	1.17	5.19	15.47	1	-0.47	0.000123	2.93	Histidine metabolism
36	2,6-Dihydroxybenzoic acid	153.0180	3.19	8.50	3.08	4	-0.61	0.013891	1.71	Others
37	β-Hydroxyisovaleric acid	117.0550	1.71	5.98	1.20	3	0.51	0.000563	2.34	Others

Abbreviations: Coefficient of variation (CV); Epithelial ovarian cancer (EOC); Fold change (FC); Measured mass to charge ratio (m/z); Retention time (min, RT); Variable importance in the projection (VIP).

[a] Metabolite identification was classed into 4 levels based on the Metabolomics Standard Initiative: 1. Metabolites are verified by reference standards. 2. Metabolites are identified based on accurate mass data, retention time, experimental MS/MS spectra, and library MS/MS spectra. 3. Metabolites are identified based on accurate mass data, retention time, experimental MS/MS spectra, and knowledge of characteristic MS/MS ions spectra. 4. Metabolites are speculated based on accurate mass data, retention time, and biological relevance.

[b] Fold change was calculated from the arithmetic mean values of each group. Fold change with a positive value indicates that the concentration of certain metabolite is up-regulated in primary EOC patients compared to controls.

[c] This ion was the fragment ion of the metabolite ([M-H2O+H]+).

[d] This ion was the adduct ion of the metabolite ([M+ACN+H]+).

Table S2 The detailed information about 30 potential biomarkers that are differential between pre- and post-operative EOC patients

No.	Metabolite	m/z	RT(min)	ppm	CV%	L [a]	FC [b]	P	VIP	Pathway
Positive-ion electrospray ionization mode (ESI+)										
1	LysoPC(22:5)	570.3534	16.53	3.51	3.16	3	-0.52	0.007033	2.11	Glycerophospholipid metabolism
2	LysoPC(20:3)	546.3544	17.07	1.83	3.27	3	-0.52	0.020812	1.82	Glycerophospholipid metabolism
3	LysoPC(20:4)	544.3369	15.83	5.33	3.16	3	-0.43	0.007716	2.09	Glycerophospholipid metabolism
4	Kynurenine	209.0914	3.35	3.35	11.19	1	-0.38	0.003527	2.31	Tryptophan metabolism
5	3-Indolepropionic acid	190.0855	10.28	4.21	5.73	1	-3.27	0.00016	2.92	Tryptophan metabolism
6	Piperine	286.1431	13.84	2.45	2.89	1	-3.2	0.000275	2.91	Lysine metabolism
7	L-Valine	118.0857	1.32	5.08	17.34	1	0.42	0.004208	2.25	Valine metabolism
8	3,4-Dihydroxymandel aldehyde	169.0490	9.93	2.96	6.89	4	-1.72	0.025248	1.80	Tyrosine metabolism
9	Creatine	132.0768	1.17	0.00	8.02	2	0.71	0.000211	2.88	Arginine and Proline Metabolism
10	Creatinine	114.0661	1.17	0.88	2.31	2	-0.29	0.001363	2.49	Arginine and Proline Metabolism
11	Uric acid	169.0355	1.38	0.59	14.93	1	-0.47	0.004101	2.24	Purine metabolism
12	Glycocholic Acid	466.3149	11.14	3.00	9.26	2	1.15	0.015622	1.91	Bile Acid Biosynthesis
13	24,25-Hydroxyvitamin D3	417.3343	11.16	4.79	15.76	4	0.84	0.001372	2.48	Steroid biosynthesis
14	α-Linolenic Acid	279.2312	19.37	2.51	1.55	2	0.43	0.023751	1.78	Linoleic acid metabolism
Negative-ion electrospray ionization mode (ESI-)										
15	Capric acid	171.1378	15.67	7.60	7.51	4	2.04	0.007265	1.58	Fatty acid biosynthesis
16	Caprylic acid	143.1069	13.00	6.29	10.07	4	1.9	0.030718	1.29	Fatty acid biosynthesis
17	19,20-DiHDPA	361.2359	14.61	6.92	6.87	3	0.45	0.002449	1.78	Fatty acid metabolism
18	12,13-DiHOME	313.2356	20.03	8.94	6.24	3	1.27	0.000198	2.25	Linoleic acid metabolism
19	Glyceric acid	105.0198	1.27	4.76	9.10	2	0.29	0.023953	1.45	Glycerolipid Metabolism
20	γ-CEHC	263.1273	10.80	6.08	4.72	2	0.78	0.006541	1.94	Vitamin E metabolism
21	Alpha-CEHC	277.1419	15.44	9.38	8.26	2	1.98	0.002141	1.79	Vitamin E metabolism
22	p-Cresol sulfate	187.0053	3.33	9.63	11.94	2	-4.3	7.24E-07	2.87	Microbial metabolism
23	Indoxylsulfuric acid	212.0007	2.21	7.55	3.77	2	-1.45	0.013339	1.56	Tryptophan metabolism
24	Hydroxyphenyllactic acid	181.0497	1.46	4.97	2.32	2	-0.37	0.001093	1.84	Tyrosine metabolism
25	Pyroglutamic acid	128.0352	1.17	0.78	1.66	2	0.30	0.044838	1.18	Glutathione Metabolism
26	L-beta-aspartyl-L-	261.0736	10.62	3.06	5.51	4	-0.67	0.004522	1.72	Protein breakdown

	glutamic acid									
27	L-Lactic acid	89.0249	1.44	5.62	2.85	1	-0.24	0.011231	1.51	Glycolysis Pyruvate Metabolism
28	Perillic acid	165.0913	12.49	4.85	4.47	4	0.46	0.018894	1.51	Limonene and pinene degradation
29	3-Dehydroquinic acid	189.0402	1.16	1.59	8.03	4	0.49	0.001602	1.83	Others
30	2,6-Dihydroxybenzoic acid	153.0180	3.187	8.50	3.08	4	-0.69	0.037298	1.38	Others

[a] Metabolite identification was classed into 4 levels based on the Metabolomics Standard Initiative (Same as Table S1).

[b] Fold change was calculated from the arithmetic mean values of each group. Fold change with a positive value indicates that the concentration of certain metabolite is up-regulated in post-operative EOC patients compared to primary EOC patients.

Table S3 The detailed information about 42 potential biomarkers that are differential between post-operative EOC patients and relapsed EOC patients

No.	Metabolite	m/z	RT(min)	ppm	CV%	L [a]	FC [b]	P	VIP	Pathway
Positive-ion electrospray ionization mode (ESI+)										
1	LysoPC(14:0)	468.3073	14.61	2.56	4.54	3	1.25	9.09E-07	2.45	Glycerophospholipid metabolism
2	LysoPC(20:3)	546.3544	17.07	1.83	3.27	3	0.92	0.000175	1.99	Glycerophospholipid metabolism
3	LysoPC(16:1)	494.3232	15.26	1.82	4.26	3	0.79	0.00027	1.96	Glycerophospholipid metabolism
4	LysoPC(15:0)	482.3277	15.91	7.46	4.73	3	0.65	0.000312	1.88	Glycerophospholipid metabolism
5	LysoPC(22:5)	570.3534	16.53	3.51	3.16	3	0.71	0.000659	1.89	Glycerophospholipid metabolism
6	LysoPC(16:0)	496.3368	17.65	6.04	2.52	1	0.37	0.004413	1.70	Glycerophospholipid metabolism
7	LysoPC(18:2)	520.3366	16.04	6.15	2.69	3	0.49	0.004159	1.72	Glycerophospholipid metabolism
8	LysoPC(18:3)	518.3227	14.76	2.70	4.16	3	0.80	0.011526	1.53	Glycerophospholipid metabolism
9	LysoPC(20:4)	544.3369	15.83	5.33	3.16	3	0.41	0.012363	1.61	Glycerophospholipid metabolism
10	LysoPE(16:1/0:0)	452.2763	14.21	1.99	4.02	3	0.99	0.00039	1.90	Lysophospholipid metabolism
11	LysoPE(18:2/0:0)	478.293	14.86	0.42	3.76	3	0.61	0.00141	1.82	Lysophospholipid metabolism
12	LysoPE(0:0/22:5)	528.3069	15.28	3.03	4.36	3	0.40	0.009183	1.64	Lysophospholipid metabolism
13	LysoPE(0:0/22:4）	530.3232	16.34	1.70	3.60	3	0.51	0.01158	1.44	Lysophospholipid metabolism
14	LysoPE(0:0/20:5)	500.2764	13.95	-1.60	4.41	3	0.57	0.014937	1.59	Lysophospholipid metabolism
15	Creatinine	114.0661	1.17	0.88	2.31	2	0.35	9.18E-05	2.28	Arginine and Proline Metabolism
16	Creatine	132.0768	1.17	0.00	8.02	2	-0.80	0.000306	1.90	Arginine and Proline Metabolism
17	L-Tryptophan	205.0957	5.53	7.31	9.65	1	0.30	0.009646	1.52	Tryptophan metabolism
18	3-Indolepropionic acid	190.0855	10.28	4.21	5.73	1	4.64	1.91E-05	2.62	Tryptophan metabolism
19	L-Hexanoylcarnitine	260.1847	7.69	3.46	13.58	3	-0.69	0.004253	1.57	Fatty acid oxidation
20	4,8 dimethylnonanoyl carnitine	330.2623	5.50	4.84	4.04	3	0.78	0.000972	2.03	Fatty acid oxidation
21	2,6 dimethylheptanoyl	302.2309	10.47	5.62	10.52	3	0.70	0.002046	1.81	Fatty acid oxidation

	carnitine									
22	Hypoxanthine	137.0460	1.43	1.46	2.41	1	-0.84	0.001802	1.79	Purine metabolism
23	Piperine	286.1431	13.84	2.45	2.89	1	3.40	0.000794	1.98	Lysine metabolism
24	MG(24:6/0:0/0:0)	431.3139	16.63	3.94	4.66	4	0.28	0.008309	1.42	Monoacylglyceride metabolism
25	2-octenoylcarnitine	286.2005	8.89	2.80	3.91	3	-0.75	0.001258	1.85	Others
Negative-ion electrospray ionization mode (ESI-)										
26	LysoPE(0:0/18:2)	476.2748	14.84	7.35	3.54	3	0.61	0.002224	1.58	Lysophospholipid metabolism
27	LysoPE(20:5/0:0)	498.2587	13.95	7.83	6.70	3	0.67	0.016957	1.29	Lysophospholipid metabolism
28	p-Cresol sulfate	187.0053	3.33	9.63	11.94	2	5.25	0.000244	1.99	Microbial metabolism
29	Indoxylsulfuric acid	212.0007	2.21	7.55	3.77	2	1.62	0.016512	1.23	Tryptophan metabolism
30	Hydroxyphenyllactic acid	181.0497	1.46	4.97	2.32	2	0.35	0.002346	1.54	Tyrosine metabolism
31	Pyroglutamic acid	128.0352	1.17	0.78	1.66	2	-0.42	0.018433	1.22	Glutathione Metabolism
32	Uric acid	167.0207	1.33	2.39	3.11	1	0.38	6.59E-05	2.02	Purine metabolism
33	12,13-DiHODE	311.2202	13.38	8.35	4.07	3	1.02	3.20E-07	2.63	Fatty acid metabolism
34	L-Glutamine	145.0619	1.15	0.00	4.53	2	0.42	0.001521	1.63	Purine metabolism
35	L-Histidine	154.0614	1.17	5.19	15.47	1	0.37	0.002173	1.57	Histidine metabolism
36	p-Salicylic acid	137.0242	2.74	1.46	14.97	2	1.32	0.005454	1.48	Ubiquinone biosynthesis
37	Capric acid	171.1378	15.67	7.60	7.51	4	-2.00	0.007919	1.39	Fatty acid biosynthesis
38	Caprylic acid	143.1069	13.00	6.29	10.07	4	-1.78	0.03131	1.13	Fatty acid biosynthesis
39	2-Octenoic acid	141.0910	11.15	7.80	10.89	2	-0.58	0.014356	1.36	Fatty acid biosynthesis
40	19,20-DiHDPA	361.2359	14.61	6.92	6.87	3	-0.40	0.005663	1.58	Fatty acid metabolism
41	2,6-Dihydroxybenzoic acid	153.0180	3.19	8.50	3.08	4	1.20	8.07E-05	1.95	Others
42	β-Hydroxyisovaleric acid	117.0550	1.71	5.98	1.20	3	-0.38	0.01477	1.26	Others

[a] Metabolite identification was classed into 4 levels based on the Metabolomics Standard Initiative (Same as Table S1).

[b] Fold change was calculated from the arithmetic mean values of each group. Fold change with a positive value indicates that the concentration of certain metabolite is up-regulated in recurrent EOC patients compared to post-operative EOC patients.

Table S4 The detailed information about 26 potential biomarkers that are differential between primary EOC patients and relapsed EOC patients

No.	Metabolite	m/z	RT(min)	ppm	CV%	L [a]	FC [b]	P	VIP	Pathway
Positive-ion electrospray ionization mode (ESI+)										
1	LysoPC(14:0)	468.3073	14.61	2.56	4.54	3	1.04	1.57E-06	3.53	Glycerophospholipid metabolism
2	LysoPC(18:3)	518.3227	14.76	2.70	4.16	3	0.89	0.00094	2.56	Glycerophospholipid metabolism
3	LysoPC(18:2)	520.3366	16.04	6.15	2.69	3	0.28	0.033342	1.65	Glycerophospholipid metabolism
4	LysoPC(16:1)	494.3232	15.26	1.82	4.26	3	0.6	0.000278	2.73	Glycerophospholipid metabolism
5	LysoPC(20:3)	546.3544	17.07	1.83	3.27	3	0.4	0.028916	1.69	Glycerophospholipid metabolism
6	LysoPE(18:2/0:0)	478.293	14.86	0.42	3.76	3	0.41	0.010696	1.96	Lysophospholipid metabolism
7	LysoPE(0:0/18:1)	480.3082	16.16	0.62	1.90	3	0.31	0.029878	1.68	Lysophospholipid metabolism
8	Methyl jasmonate	225.1490	15.57	2.22	3.79	4	0.72	0.0071	2.11	α-Linolenic acid metabolism
9	Tetracosahexaenoic acid	357.2776	14.80	3.36	9.68	3	1.28	0.039796	1.60	Linoleic acid metabolism
10	MG(24:6/0:0/0:0)	431.3139	16.63	3.94	4.66	4	0.43	0.000181	2.80	Monoacylglycerol metabolism
11	L-Tryptophan	205.0957	5.53	7.31	9.65	1	0.32	0.000786	2.54	Tryptophan metabolism
12	L-Kynurenine	209.0914	3.35	3.35	11.19	1	-0.38	0.015785	1.86	Tryptophan metabolism
13	3-Indolepropionic acid	190.0855	10.28	4.21	5.73	1	1.38	0.007036	2.09	Tryptophan metabolism
14	Hypoxanthine	137.046	1.43	1.46	2.41	1	-0.85	0.002082	2.34	Purine metabolism
15	Retinol	269.2253	20.44	5.94	6.53	2	0.38	0.006959	2.08	Retinol metabolism
Negative-ion electrospray ionization mode (ESI-)										
16	2,3-Dihydroxyvaleric acid	133.0501	1.44	3.76	5.02	4	0.55	0.000201	2.23	Valine, leucine and isoleucine biosynthesis
17	α-Ketoisovaleric acid	115.0401	1.47	0.00	2.15	2	0.17	0.029196	1.34	Valine, Leucine and Isoleucine Degradation
18	Glyceric acid	105.0198	1.27	4.76	9.10	2	0.48	0.000241	2.18	Glycine and Serine Metabolism
19	L-Histidine	154.0614	1.17	5.19	15.47	1	0.57	0.000146	2.62	Histidine metabolism
20	L-Arabinonic acid	165.0395	1.31	6.06	2.82	4	0.34	0.018832	1.64	Ascorbate and aldarate metabolism
21	Purine	119.0351	1.19	0.84	2.08	1	0.33	0.035437	1.33	Purine metabolism
22	p-Salicylic acid	137.0242	2.74	1.46	14.97	2	0.91	0.020158	1.50	Ubiquinone biosynthesis

23	Nutriacholic acid	389.2665	12.91	8.22	8.76	4	0.63	0.003105	1.87	Bile acid metabolism
24	12,13-DiHODE	311.2200	13.38	9.00	4.07	3	0.83	4.01E-05	2.61	Fatty acid metabolism
25	γ-CEHC	263.1273	10.80	6.08	4.72	2	0.77	0.009211	1.59	Vitamin E metabolism
26	4-O-Methylgallic acid	183.0287	4.36	6.56	7.35	4	-1.01	0.04182	1.45	Others

[a] Metabolite identification was classed into 4 levels based on the Metabolomics Standard Initiative (Same as Table S1).

[b] Fold change was calculated from the arithmetic mean values of each group. Fold change with a positive value indicates that the concentration of certain metabolite is up-regulated in recurrent EOC patients compared to primary EOC patients.

Table S5 Changes of 37 primary EOC biomarkers in response to surgery and relapse.

Metabolite	Primary EOC *versus* control		Post-operative EOC *versus* primary EOC		Relapsed EOC *versus* post-operative EOC		Relapsed EOC *versus* primary EOC	
	FC[1]	P value	FC[2]	P value	FC[3]	P value	FC[4]	P value
2-Octenoylcarnitine	**-0.83**	**0.000109**	**0.85**	**0.000211**	**-0.75**	**0.001258**	0.09	0.434425
2-Octenoic acid	**-0.77**	**0.001108**	**0.85**	**0.00028**	**-0.81**	**0.000983**	0.03	0.636167
Hydroxyphenyllactic acid	**0.22**	**0.013832**	**-0.37**	**0.001093**	**0.35**	**0.002346**	-0.02	0.841128
19,20-DiHDPA	**-0.36**	**0.001648**	**0.45**	**0.002449**	**-0.40**	**0.005663**	0.04	0.608742
Retinol	**-0.47**	**0.001617**	**0.37**	**0.006782**	0.00	0.77306	**0.38**	**0.006959**
Kynurenine	**0.59**	**0.00039**	**-0.38**	**0.003527**	0.00	0.639322	**-0.38**	**0.015785**
Dodecanedioic acid	**-0.66**	**0.00119**	**0.58**	**0.001172**	0.09	0.226523	**0.67**	**0.003711**
γ-CEHC	**-0.95**	**0.008108**	**0.78**	**0.006541**	-0.01	0.353464	**0.77**	**0.009211**
LysoPC(22:5)	**-0.57**	**0.002168**	**-0.52**	**0.007033**	**0.71**	**0.000659**	0.20	0.167051
LysoPC(20:4)	**-0.43**	**0.000748**	**-0.43**	**0.007716**	**0.41**	**0.012363**	-0.02	0.985453
Piperine	**-2.37**	**0.015023**	**-3.20**	**0.000275**	**3.40**	**0.000794**	0.20	0.709247
LysoPC(20:3)	**-0.58**	**0.002075**	**-0.52**	**0.020812**	**0.92**	**0.000175**	**0.40**	**0.028916**
3-Indolepropionic acid	**-1.47**	**0.005872**	**-3.27**	**0.00016**	**4.64**	**1.91E-05**	**1.38**	**0.007036**
2,6-Dihydroxybenzoic acid	**-0.61**	**0.013891**	**-0.69**	**0.037298**	**1.20**	**8.07E-05**	**0.51**	**0.014355**
LysoPC(18:2)	**-0.60**	**3.20E-06**	-0.21	0.301223	**0.49**	**0.004159**	**0.28**	**0.033342**
LysoPC(18:3)	**-0.83**	**0.00018**	0.09	0.347757	**0.8**	**0.011526**	**0.89**	**0.00094**
LysoPC(14:0)	**-0.85**	**0.00019**	-0.21	0.607435	**1.25**	**9.09E-07**	**1.04**	**1.57E-06**
LysoPC(16:1)	**-0.6**	**0.000806**	-0.20	0.599956	**0.79**	**0.00027**	**0.60**	**0.000278**
LysoPE(18:2/0:0)	**-0.52**	**0.002168**	-0.20	0.360969	**0.61**	**0.00141**	**0.41**	**0.010696**
MG(24:6/0:0/0:0)	**-0.39**	**0.0014**	0.15	0.229719	**0.28**	**0.008309**	**0.43**	**0.000181**
L-Tryptophan	**-0.32**	**0.011869**	0.02	0.605367	**0.30**	**0.009646**	**0.32**	**0.000786**
L-Histidine	**-0.47**	**0.000123**	0.19	0.196602	**0.37**	**0.002173**	**0.57**	**0.000146**
p-Salicylic acid	**-1.1**	**0.002942**	-0.41	0.079453	**1.32**	**0.005454**	**0.91**	**0.020158**
12,13-DiHODE	**-0.74**	**0.000215**	-0.19	0.233109	**1.02**	**3.20E-07**	**0.83**	**4.01E-05**
Hypoxanthine	**0.85**	**0.004392**	-0.01	0.701204	**-0.84**	**0.001802**	**-0.85**	**0.002082**
LysoPC(18:1)	**-0.44**	**0.001012**	-0.05	0.914828	0.25	0.2475	0.20	0.139409
2-Piperidinone	**-0.61**	**0.004895**	-0.14	0.882196	0.38	0.102403	0.25	0.092181
Glycoursodeoxycholic acid	**-1.80**	**0.001835**	-0.58	0.922145	1.17	0.194567	0.59	0.139253
Aldosterone	**-0.58**	**9.51E-05**	0.19	0.145546	-0.24	0.640992	-0.05	0.434055
Deoxycholic acid	**-1.11**	**0.013663**	-1.01	0.969167	1.46	0.605525	0.46	0.405786
N-Undecanoylglycine	**0.32**	**0.021176**	0.06	0.84345	-0.05	0.99857	0.01	0.860885
4-Nitrophenol	**0.24**	**0.002721**	-0.11	0.156768	0.03	0.625363	-0.08	0.355364
L-Thyroxine	**0.25**	**0.006899**	-0.05	0.971279	0.04	0.85879	-0.01	0.875616
tetracosahexaenoic acid	**-1.86**	**0.003231**	0.12	0.183608	1.16	0.801992	**1.28**	**0.039796**
LysoPE(0:0/18:1)	**-0.37**	**0.008377**	0.03	0.581991	0.28	0.154161	**0.31**	**0.029878**
Nutriacholic acid	**-0.6**	**0.000338**	-0.24	0.418741	0.87	0.064167	**0.63**	**0.003105**
β-Hydroxyisovaleric acid	**0.51**	**0.000563**	0.14	0.30018	**-0.38**	**0.01477**	-0.24	0.067474

[1]Fold change with a positive value indicates that the concentration of certain metabolite is up-regulated in primary EOC patients compared to controls.

[2]Fold change with a positive value indicates that the concentration of certain metabolite is up-regulated in post-operative EOC patients compared to primary EOC patients.

[3]Fold change with a positive value indicates that the concentration of certain metabolite is up-regulated in recurrent EOC patients compared to post-operative EOC patients.

[4]Fold change with a positive value indicates that the concentration of certain metabolite is up-regulated in recurrent EOC patients compared to primary EOC patients.

Welch’s t test was performed to determine the statistical significance of metabolite difference between groups, and P values under 0.05 were marked in bold.
